# Protocol for phenotyping mouse myeloid and lymphoid cells by mass cytometry

**DOI:** 10.1016/j.xpro.2025.103684

**Published:** 2025-03-10

**Authors:** Laura Morin, Brice Autier, Patrice Hemon, Simon Le Gallou, Mikael Roussel, Sarah Dion, Valérie Lecureur

**Affiliations:** 1 University Rennes, INSERM, EHESP, IRSET (Institut de recherche en santé, environnement et travail) – UMR_S 1085, 35000 Rennes, France; 2 University Rennes, CHU Rennes, INSERM, EHESP, IRSET (Institut de recherche en santé, environnement et travail) – UMR_S 1085, 35000 Rennes, France; 3 LBAI, INSERM UMR1227, University of Brest, 29200 Brest, France; 4 Centre Hospitalier Universitaire de Rennes, Pôle Biologie, Rennes, France; 5 Institut National de la Santé et de la Recherche Médicale, Unité Mixte de Recherche U1236, Université Rennes 1, Etablissement Français du Sang Bretagne, Rennes, France

**Keywords:** cell isolation, single cell, mass cytometry

## Abstract

Cytometry by time of flight (CyTOF) is a flow cytometry-based technique using metal-tagged antibodies, allowing immunophenotyping. Here, we present a protocol for phenotyping mouse myeloid and lymphoid cells isolated from lung, spleen, and intraperitoneal lavages in healthy or pathologic conditions. We describe steps for antibody labeling and titration, tissue dissociations, and staining. We then detail procedures to compare staining obtained from fresh or cryopreserved tissues.

For complete details on the use and execution of this protocol, please refer to Morin et al.^1^

## Highlights

• Protocol to phenotype mouse myeloid and lymphoid cells by mass cytometry
• Antibody labeling to heavy metals and titration of a 37-antibody panel
• Steps to prepare single-cell suspension from lung and spleen tissues
• Cell staining, sample acquisition, data cleaning, and cell gating strategy

## Abstract (editor highlights)

Publisher’s note: Undertaking any experimental protocol requires adherence to local institutional guidelines for laboratory safety and ethics.

## Graphical abstract

**Figure:**
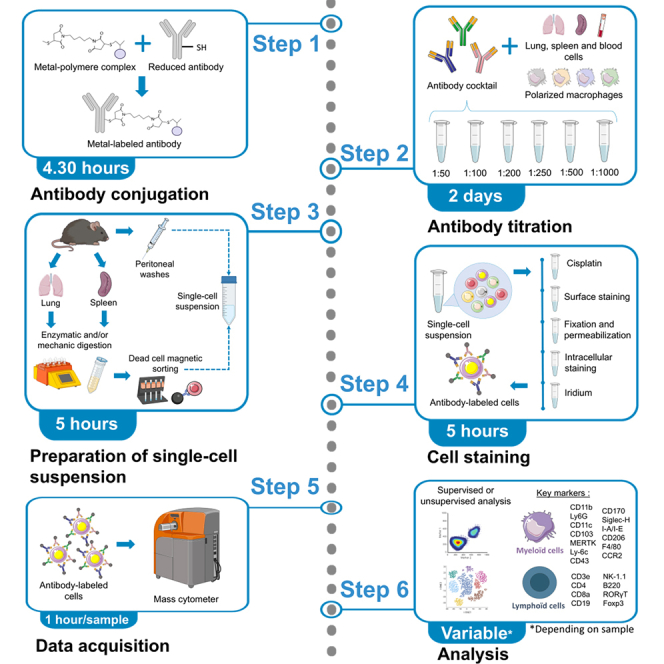

## Before you begin

Immune cells are key players in the defense against pathogens and the maintenance of tissue homeostasis. These cell populations are highly complex and heterogeneous, so more powerful single-cell techniques are needed to characterize them. Mass cytometry (Cytometry by Time of Flight, CyTOF) allows single-cell phenotyping and quantification analyses of numerous cell types simultaneously using up to 50 antibodies by panel.^2^ The protocol below aims to identify lymphoid and myeloid cells in various healthy or pathologic tissues and fluids such as lung, spleen and intraperitoneal lavage. A 37-antibody panel is presented to identify macrophage, dendritic cell, granulocyte and T and B cell sub-populations with a focus made on myeloid cells by using several macrophage tissue-specific markers and polarization markers. Dissociation protocols are optimized for cell yield and viability depending on the tissue. We also analyzed the impact of tissue thawing on cell staining and identification.

### Institutional permissions

This protocol uses cells from mouse blood, lung, spleen and intraperitoneal lavages. All animal experiments were approved by the Committee on the Ethics of Animal Experiments under the French Ministry of Higher Education and Research. Please note that ethical permissions should be obtained by ethical committee of animal experimentation to perform this protocol with mouse samples (APAFIS#17011; APAFIS#28586-202011261535409).

**Table 1:** Surface and intracellular antibody cocktails for antibody titrations

	Marker	Metal	Type	Cocktail 1	Cocktail 2	Cocktail 3	Cocktail 4	Cocktail 5	Cocktail 6
**Surface antibody cocktails**	CD45	89Y	Standard BioTools	x					
	CD204	116Cd	Custom	x					
	Ly-6G	141Pr	Standard BioTools			x			
	CD11c	142Nd	Standard BioTools		x				
	CD115	144Nd	Standard BioTools	x					
	CD4	145Nd	Standard BioTools			x			
	CD43	146Nd	Standard BioTools				x		
	CD103	147Sm	Custom	x					
	CD11b	148Nd	Standard BioTools					x	
	CD19	149Sm	Standard BioTools		x				
	CD24	150Nd	Standard BioTools						x
	CD64	151Eu	Standard BioTools	x					
	CD3e	152Sm	Standard BioTools						x
	CD8a	153Eu	Standard BioTools			x			
	MERTK	156Gd	Custom	x					
	CCR2	158Gd	Custom					x	
	F4/80	159Tb	Standard BioTools						x
	TREM-2	160Gd	Custom			x			
	Ly-6C	162Dy	Standard BioTools			x			
	CD326	163Dy	Custom		x				
	CX3CR1	164Dy	Standard BioTools	x					
	CD31	165Ho	Standard BioTools				x		
	Gp-38	167Er	Custom		x				
	CD169	170Er	Standard BioTools						x
	CD172a	171Yb	Custom		x				
	CD170	172Yb	Custom						x
	NK-1.1	173Yb	Custom			x			
	Siglec H	174Yb	Custom				x		
	CD45R	176Yb	Standard BioTools					x	
	I-A/I-E	209Bi	Standard BioTools						x
**Intra-cellular antibody cocktails**	CD68	154Sm	Custom	x					
	RORγT	155Gd	Custom			x			
	iNOS	161Dy	Standard BioTools					x	
	FOXP3	166Er	Custom			x			
	CD209a	168Er	Custom	x					
	ARG-1	169Tm	Custom					x	
	CD206	175Lu	Custom		x

**Figure 1:**
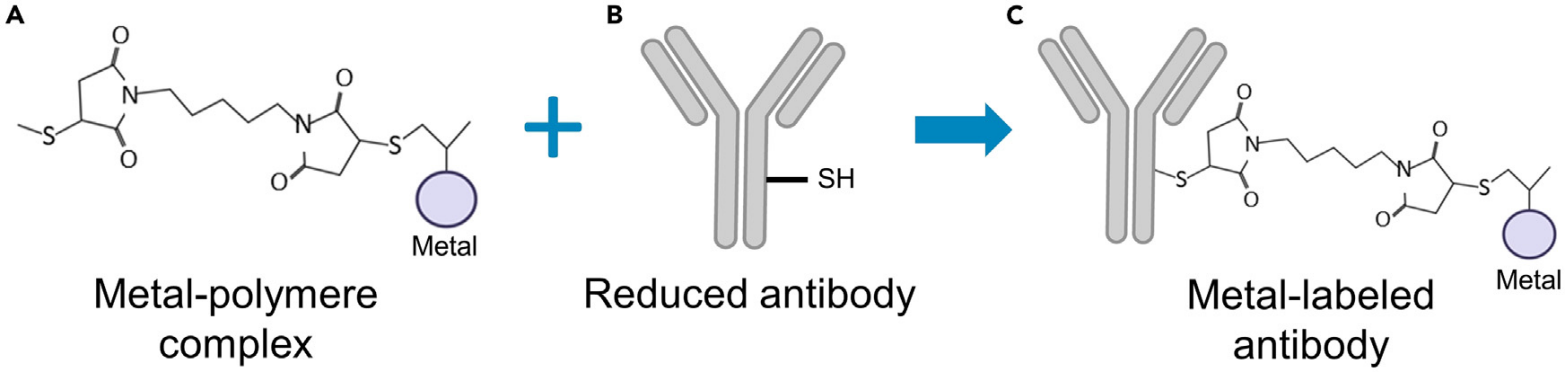
Antibody-metal conjugation procedure (adapted from Standard BioTools protocol) (A) Metal-loaded polymer. (B) Reduced antibody. (C) Antibody conjugated to metal-loaded polymer.

### Antibody conjugation to lanthanide metals

**Timing: 4 h 30 min**

**CRITICAL:** To avoid metal contaminations, only use pipette tips with filter and non-autoclaved materials. troubleshooting 1.

***Note:*** The following protocol describes the antibody conjugation to lanthanide metals using the Maxpar X8 antibody labeling kit. Buffers used in this protocol (L, R, W and C buffers), Maxpar X8 polymers and metals are part of this kit. The antibody stock solutions and filters used in this protocol are not included in the Maxpar X8 antibody labeling kit and need to be purchased separately.

***Note:*** The conjugation of antibodies to cadmium metals requires the use of the Maxpar MCP9 antibody labeling kit. This protocol is not described below, please refer to user guide of the kit: link.

***Note:*** Each antibody needs to be conjugated with a metal. Steps will need to be repeated for each antibody. Suggested antibody-metal conjugations are listed in Table 1.

1. Load the metal to the Maxpar X8 polymer for lanthanides (Figure 1A).
  ***Note:*** The MAXPAR Polymer is moisture-sensitive. Equilibrate Polymer (stored at −20°C) to room temperature before opening to avoid moisture condensation.
  a. Centrifuge the Maxpar X8 polymer tube 10 s in micro-centrifuge and resuspend it into 95 μL of L-Buffer.
  b. Add 5 μL of the 50 mM metal stock solution (Maxpar X8 antibody labeling kit) in the Maxpar X8 polymer tube in order to have a final concentration of 2.5 mM in 100 μL.
  c. Mix several times with a pipette and incubate the solution at 37°C in water bath for 30 min.
    **CRITICAL:** Do not exceed 40 min incubation time.
    ***Note:*** While in incubation, proceed to reducing antibody.
2. Reduce antibody (Figure 1B).
  a. Spin the stock antibody solution with microcentrifuge at 700 × *g* for 30 s.
  b. Place a 50 kDa filter in a collector tube and add 100 μg of antibody to the filter.
  c. If necessary, add the required volume of R-buffer in the filtration unit to reach a final volume of 400 μL.
    ***Note:*** The stock antibody solution must not contain bovine serum albumin (BSA).
    ***Note:*** If the stock antibody volume is higher than 400 μL, it can be pre-concentrated. Add 400 μL of stock antibody solution to the 50 kDa filter and centrifuge at 14 000 × *g* for 10 min at room temperature. Then add the remaining volume of antibody stock solution to the 50 kDa filtration unit and adjust the volume to 400 μL with R-buffer.
  d. Centrifuge at 14 000 × *g* for 10 min at room temperature.
  e. Add 400 μL of R-buffer to 50 kDa filter to wash it.
  f. Centrifuge at 14 000 × *g* for 10 min at room temperature.
  g. Dilute the 0.5 M TCEP (tris(2-carboxyethyl)phosphine) stock solution in R-buffer to obtain a final concentration of 4 mM (100 μL of this solution is required for each antibody conjugation).
  h. Discard the eluate obtained after centrifugation in the collector tube and place the 50 kDa filter in this same flow-through tube.
  i. Add 100 μL of the 4 mM TCEP solution in the filtration unit and mix using a pipette.
  j. Incubate the solution at 37°C in water bath for 30 min.
    **CRITICAL:** Do not exceed 30 min incubation time.
3. Purify the metal-loaded polymers.
  a. Place a 3 kDa filter in collector tube and add 200 μL of the L-buffer to the filter.
  b. Add 100 μL of metal-loaded polymer (from Step 1.c.) to the 3 kDa filter containing 200 μL of L-buffer.
  c. Centrifuge at 14 000 × *g* for 25 min at room temperature.
  d. Discard the eluate and add 400 μL of C-buffer in the filtration unit.
  e. Centrifuge at 14 000 × *g* for 30 min at room temperature.
4. Purify the partially reduced antibody.
  a. Remove the 50 kDa filter containing antibody from the water bath.
  b. Wash antibodies by adding 300 μL of C-buffer in the filtration unit.
  c. Centrifuge at 14 000 × *g* for 10 min at room temperature.
  d. Discard eluate and add 400 μL of C-buffer in filter.
  e. Centrifuge at 14 000 × *g* for 10 min at room temperature.

***Note:*** Two centrifuges can be used to perform steps 3 and 4 simultaneously.

5. Recover the purified metal-loaded polymers and reduced antibodies.
  a. Recover the 3 kDa filter containing the purified metal-loaded polymers and discard eluate.
  b. Recover the 50 kDa filter containing the purified reduced antibodies and discard eluate.
6. Conjugate reduced antibodies with metal-loaded polymers (Figure 1C).
  a. Evaluate post-centrifugation residual volume present in 3 kDa filter using a pipette.
  b. Resuspend the polymers in 3 kDa filter with C-buffer to have a final volume of 80 μL.
  c. Transfer 80 μL of polymer solution to the 50 kDa filter containing the antibody for a final volume of 100 μL (approximatively 20 μL of residual volume).
  d. Mix gently using a pipette and rinsing the filter walls.
  e. Incubate at 37°C in water bath for 90 min.
7. Wash the conjugate antibodies.
  a. Add 200 μL of W-buffer to the 50 kDa filter containing 100 μL of conjugate antibodies.
  b. Centrifuge at 14 000 × *g* for 10 min at room temperature.
  c. Discard eluate.
  d. Add 400 μL of W-buffer to the 50 kDa filter.
  e. Centrifuge at 14 000 × *g* for 10 min at room temperature.
  f. Discard eluate.
  g. Repeat the wash two more times.
8. Evaluate the yield.
  a. Evaluate the residual volume post-centrifugation using a pipette.
  b. Add the necessary volume of W-buffer in the 50 kDa filter to obtain a final volume of 101.5 μL.
  c. Mix gently using a pipette.
  d. Quantify antibody yield by measuring protein absorbance at 280 nm on 1.5 μL using NanoDrop, the blank is done with W-buffer.
  e. From the amount of protein measured by the NanoDrop in μg/μL, the yield can be calculated using these equations:

Antibodymassrecover(μg)=Finalantibodyconcentration(μg/μl)×finalvolume(100μl)

$$Antibody\;yield\;\left(\%\right)=\frac{Antibody\;mass\;recover\;\left(\mu g\right)}{Initial\;antibody\;mass\;\left(=100\;\mu g\right)}\times 100$$

***Note:*** The antibody yield is often comprised between 60 and 95%.

9. Recover and store conjugate antibody.
  a. Centrifuge the 50 kDa filter at 14 000 × *g* for 10 min at room temperature to remove W-buffer.
  b. Evaluate post-centrifugation residual volume present in 50 kDa filter using a pipette.
  c. Calculate the necessary volume of Antibody Stabilizer solution Candor to add for a final antibody concentration of 0.5 mg/mL using this equation:
    $$Stabilizer\;solution\;\left(ml\right)=\frac{Antibody\;mass\;recover\;\left(\mu g\right)\times 1\;ml}{500\;\mu g}-residual\;volume\;\left(ml\right)$$
  d. Add the calculated volume of Antibody Stabilizer solution in the 50 kDa filter in sterile conditions.
  e. Invert the filter into another labeled-recovery tube and centrifuge at 1 000 × *g* for 2 min at room temperature.
  f. Remove filter and transfer the conjugate antibodies into another storage tube with screw cap to avoid evaporation and store at 4°C.
    ***Note:*** Standard Biotools recommends that labeled antibodies are stable for 6 months after preparation. Moreover, we recommend always using stock antibody solutions under sterile conditions to avoid contamination.

### Antibody titrations and metal spillover checking

**Timing: 2 days**

***Note:*** This step takes 2 days, but additional titration tests can be required if the optimal dilution was not determined.

**Figure 2:**
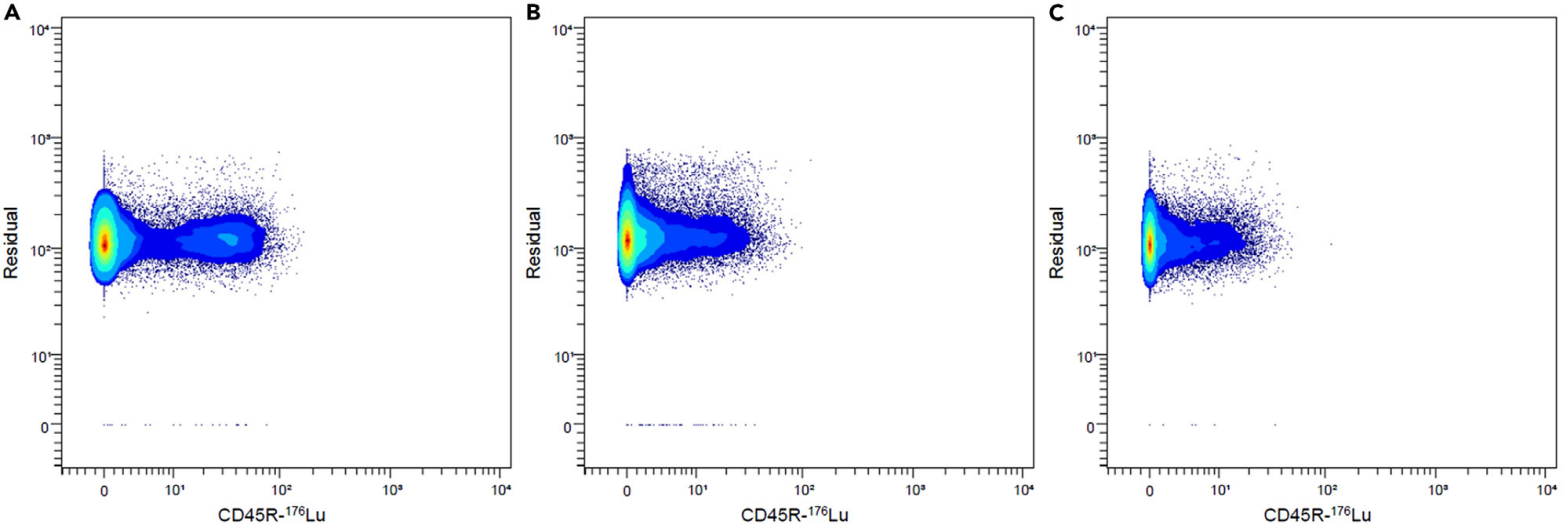
Example of antibody titration Plots showing cell staining using 1:50 (A), 1:100 (B) and 1:200 (C) dilutions of the CD45R-^176^Lu antibody (antibody cocktail 5). The used dilution was 1:50 as it was the lowest allowing good discrimination of positive and negative populations.

The antibody titration is used for the evaluation of the optimal antibody concentration to use for cell staining. Several antibody dilutions should be tested to choose the optimal dilution for differentiation of positive cells from negative cells (Figure 2). An overly low antibody concentration may not allow proper labeling, while a too high concentration could result in nonspecific labeling and signal overlap. Moreover, metal spillover should be checked (i) 16 mass units higher than the primary metal isotope (M+16), in order to detect signal overlap due to metal oxidation following air exposure, and (ii) M±1 in order to detect isotopic impurity or too high antibody concentration. For this, antibody cocktails are prepared at different dilutions, each containing metal-coupled antibodies that cannot spillover into each other’s channels (Table 1). Of note, enzymatic dissociation of tissues can affect antigens of the cell surface, reducing or severely altering the signal. For proteins expressed both intracellularly or on the cell surface, this can be overcome by cytoplasmic labeling.^3^

***Note:*** The marker NK1.1 is only found in the following mouse strains: C57BL/6, CE/J, FVB/N, MA/MyJ, NZB/-, NZW/-, SEC/-, SJL/J and ST/bJ.

***Note:*** For the titration of this antibody panel, six cocktails are prepared, and three dilutions of each cocktail are made. Therefore, 18 tubes are required for the titration.

10. Polarize the murine macrophage cell line (RAW264.7) to several different phenotypes (e.g., materials and equipment).
  a. Culture unpolarized macrophages (M0) in DMEM medium supplemented with 10% of FBS up to 80% cell confluence in 6-well culture plates.
  b. Polarize macrophages to a pro-inflammatory phenotype (M1) using 2 mL of Pro-inflammatory M1 macrophage polarization solution per well of 6-well culture plate for 24 h.
  c. Polarize macrophages to an alternative phenotype M2a or M2c using 2 mL of Alternative M2a or M2c macrophage polarization solution per well of 6-well culture plate for 24 h.

***Note:*** A total of 4 million RAW264.7 macrophages of each phenotype (M0, M1, M2a, M2c) are required to ensure there are enough cells for the 18 tubes needed for titration.

**Figure 3:**
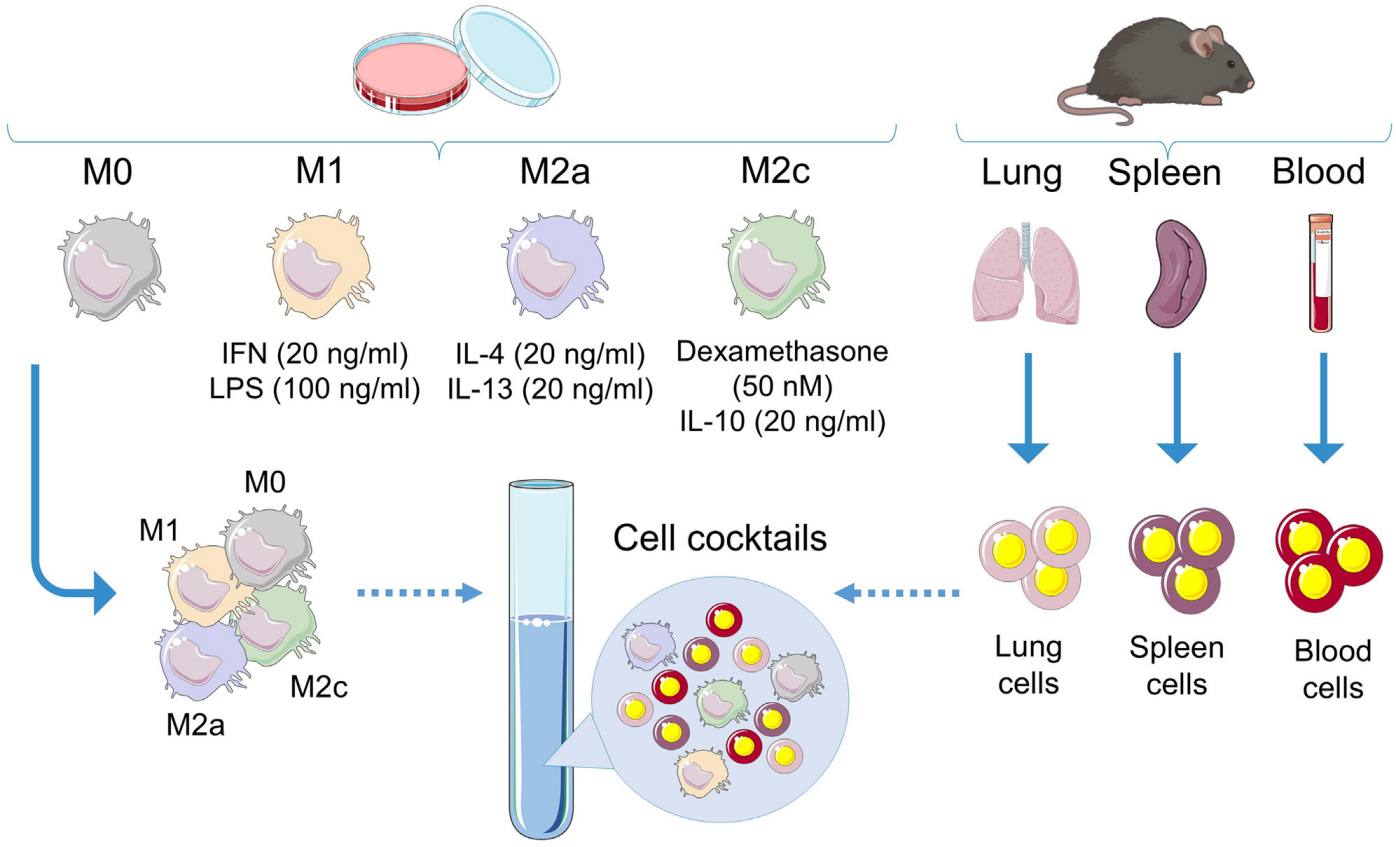
Preparation of cell cocktails for titrations using RAW 264.7 macrophage cell line and mouse tissue and fluid cells Non-polarized macrophages (M0) were cultured in DMEM medium supplemented with 10% of FBS. Polarized macrophages were cultured in presence of cytokine cocktails for additional 24 h: interferon (IFNɤ) and lipopolysaccharide (LPS) for pro-inflammatory macrophages M1; interleukin-4 and -13 for alternatives macrophages M2a; dexamethasone and interleukin-10 for alternative macrophages M2c. The cellular cocktail is composed of RAW264.7 macrophages and cells from mouse blood, lungs, and spleen.

11. Prepare surface antibody cocktails (Table 1).
  a. Label Eppendorf tubes corresponding to several dilutions of surface antibody cocktails indicated in Table 1.
  b. Dilute each antibody to 1:125, 1:250 and 1:500 for custom antibodies (stock concentration of 0,5 mg/mL) and to 1:25, 1:50 and 1:100 for Standard BioTools antibodies in appropriate cocktail tubes (2× solution).
    ***Note:*** Dilution factors provided at these steps are related to 2× antibody cocktail solution. These cocktails will be diluted to 1:2 (Step 16.b.) to obtain 1× solution.
    ***Note:*** Pre-dilutions can be made to avoid pipetting too small volumes.
  c. Add the necessary volume of Maxpar cell Staining buffer to have a final volume of 50 μL in each cocktail tube.
12. Prepare intracellular antibody cocktails (Table 1).
  a. Label Eppendorf tubes corresponding to several dilutions of intracellular antibody cocktails indicated in Table 1.
  b. Dilute each antibody to 1:125, 1:250 and 1:500 for custom antibodies (stock concentration of 0,5 mg/mL) and to 1:25, 1:50 and 1:100 for Standard BioTools antibodies in appropriate cocktail tubes (2× solution).
  c. Add the necessary volume of 1× permeabilization buffer (Foxp3/Transcription Factor Staining Buffer Set) to have a final volume of 50 μL.
13. Prepare a cocktail of several cell types in order to find all target markers (Figure 3).
  a. Isolate cells from various mouse fresh tissues/fluids such as lung, spleen and blood.
    ***Note:*** Dissociation protocols are described in following sections.
    ***Note:*** A total of 18 million lung cells, 18 million spleen cells, and 4 million blood cells are required to ensure there are enough cells for the 18 tubes needed for titration.
  b. Recover M0, M1, M2a and M2c macrophages using Accutase stripping.
    i. Remove supernatant and wash plate with sterile PBS.
    ii. Add 1 mL of Accutase to each well of the 6-well culture plate.
    iii. Incubate plate in incubator for 10 min at 37°C.
    iv. Add an equal volume of DMEM supplemented with 10% FBS to the Accutase solution to stop its action.
    v. Recover cells.
  c. Prepare a single-cell suspension of each cell types (macrophage cell line and tissue cells) and count them on Malassez chamber.
    **Alternative**: Cells can be counted using other counting chambers, such as the Neubauer or Bürker chamber, or an automated cell counter.
  d. Label 18 Falcon tubes (15 mL) corresponding to several dilutions of antibody cocktails indicated in Table 1.
  e. Distribute 1 million lung cells, 1 million spleen cells, 0.2 million blood cells, and 0.2 million RAW264.7 macrophages of each phenotype (M0, M1, M2a, M2c) into the 18 tubes to achieve a final count of 3 million cells per tube.
  f. Add Maxpar PBS supplemented with 2% FBS in tubes to have a final volume of 2 mL.
  g. Centrifuge at 400 × *g* for 5 min at 4°C and remove supernatant.
  h. Add 2 mL of Maxpar PBS supplemented with 2% FBS in tubes.
  i. Centrifuge at 400 × *g* for 5 min at 4°C and remove supernatant.
14. Stain cells with Cisplatin following the protocol described in following section (Step 12).
15. Block Fc receptors following the protocol described in following section (Step 13).
16. Stain cells with surface antibody cocktails.
  a. Resuspend cell pellet with 40 μL of Maxpar Cell Staining buffer to have a final volume of 50 μL (10 μL of residual volume).
  b. Add 50 μL of surface antibody cocktails in appropriate tubes to have a final volume of 100 μL (1× solution).
  c. Mix gently using a pipette.
  d. Incubate for 30 min at room temperature. After 15 min of incubation, resuspend again using a pipette.
  e. Add 4 mL of Maxpar cell staining buffer to wash cells.
  f. Centrifuge at 600 × *g* for 5 min at room temperature and remove supernatant.

***Note:*** Tubes containing only surface antibody cocktails can skip steps 17 and 18 and restart at step 19.

17. Fix and permeabilize cells following the protocol described in following section (Step 15).
18. Stain cells with intracellular antibody cocktails.
  a. Add 50 μL of surface antibody cocktails in appropriate tubes to have a final volume of 100 μL.
  b. Mix gently using a P100 pipette and spin tubes using microcentrifuge for 10 s.
  c. Incubate for 30 min at room temperature. After 15 min of incubation, resuspend again using a pipette.
  d. Add 2 mL of 1× permeabilization buffer (Foxp3/Transcription Factor Staining Buffer Set) to wash cells.
  e. Centrifuge at 900 × *g* for 5 min at room temperature and remove supernatant.
  f. Repeat the two latest steps to perform a second wash.
19. Fix cells with 2.5% formaldehyde solution following the protocol described in following section (Step 17).
20. Stain cells with Cell-ID Intercalator Iridium following the protocol described in following section (Step 18).
21. Perform sample acquisition on a Helios system using CyTOF software following the protocol described in following section (sample acquisition, Step 19 to 29).

**Alternative:** The use of other mass cytometers is possible but must follow a strict protocol using internal controls as shown by M.D Leipold et al.^4^

## Key resources table

**Table:** 

REAGENT or RESOURCE	SOURCE	IDENTIFIER
**Antibodies**		

Anti-mouse Ly6G (1A8)-141Pr-100 tests	Standard BioTools	Cat#3141008B; RRID: AB_2814678
Anti-mouse CD11c (N418)-142Nd-100 tests	Standard BioTools	Cat#3142003B; RRID: AB_2814737
Anti-mouse CD115 (AFS98)-144Nd-100 tests	Standard BioTools	Cat#3144012B; RRID: AB_2895116
Anti-mouse CD4 (RM4-5)-145Nd-100 tests	Standard BioTools	Cat#3145002B; RRID: AB_2687832
Anti-mouse CD43 (S11)-146Nd-100 tests	Standard BioTools	Cat#3146009B; RRID: AB_3076461
Anti-mouse CD11b (M1/70)-148Nd-100 tests	Standard BioTools	Cat#3148003B; RRID: AB_2814738
Anti-mouse CD19 (6D5)-149Sm-100 tests	Standard BioTools	Cat#3149002B; RRID: AB_2814679
Anti-mouse CD24 (M1/69)-150Nd-100 tests	Standard BioTools	Cat#3150009B; RRID: AB_2916042
Anti-mouse CD64 (X54-5/7.1)-151Eu-100 tests	Standard BioTools	Cat#3151012B; RRID: AB_2814680
Anti-mouse CD3e (145-2C11)-152Sm-100 tests	Standard BioTools	Cat#3152004B; RRID: AB_3076460
Anti-mouse CD8a (53-6.7)-153Eu-100 tests	Standard BioTools	Cat#3153012B; RRID: AB_2885019
Anti-mouse F4/80 (BM8)-159Tb-100 tests	Standard BioTools	Cat#3159009B; RRID: AB_2811238
Anti-mouse iNOS (CXNFT)-161Dy-100 tests	Standard BioTools	Cat#3161011B; RRID: AB_2922920
Anti-mouse Ly6c (HK1.4)-162Dy-100 tests	Standard BioTools	Cat#3162014B; RRID: AB_2922921
Anti-mouse CX3CR1 (SA011F11)-164Dy-100 tests	Standard BioTools	Cat#3164023B; RRID: AB_2832247
Anti-mouse CD31/PECAM-1 (390)-165Ho-100 tests	Standard BioTools	Cat#3165013B; RRID: AB_2801434
Anti-mouse CD169/Siglec-1 (3D6.112)-170Er-100 tests	Standard BioTools	Cat#3170018B; RRID: AB_2885022
Anti-mouse CD45R/B220 (RA3-6B2)-176Yb-100 tests	Standard BioTools	Cat#3176002B; RRID: AB_2895123
Anti-mouse I-A/I-E (M5/114.15.2)-209Bi-100 tests	Standard BioTools	Cat#3209006B; RRID: AB_2885025
Anti-mouse CD45 (30-F11)-89Y-100 tests	Standard BioTools	Cat#3089005B; RRID: AB_2651152
Purified anti-mouse CD204 antibody (1F8C33)	BioLegend	Cat#154702; RRID: AB_2728216
Purified anti-mouse MERTK (Mer) antibody (2B10C42)	BioLegend	Cat#151502; RRID: AB_2566624
Purified anti-mouse TREM-2 antibody (6E9)	BioLegend	Cat#824804; RRID: AB_2922646
Purified anti-mouse CD172a (SIRPα) antibody (P84)	BioLegend	Cat#144002; RRID: AB_11203711
Purified anti-mouse CD170 (Siglec-F) antibody (S17007L)	BioLegend	Cat#155502; RRID: AB_2810420
Purified anti-mouse NK-1.1 (Maxpar Ready) antibody (PK136)	BioLegend	Cat#108743; RRID: AB_2562803
Purified anti-mouse Siglec H antibody (551)	BioLegend	Cat#129602; RRID: AB_1227757
Purified anti-mouse CD326 (Ep-CAM) (Maxpar Ready) antibody (G8.8)	BioLegend	Cat#118223; RRID: AB_2563743
Purified anti-mouse Gp-38/Podoplanin antibody (8.1.1)	BioLegend	Cat#127402; RRID: AB_1089187
Purified anti-mouse CD209a (DC-SIGN) antibody (MMD3)	BioLegend	Cat#833001; RRID: AB_2564962
Purified anti-mouse CD68 antibody (FA-11)	BioLegend	Cat#137002; RRID: AB_2044003
Purified anti-mouse CD206 (MMR) antibody (C068C2)	BioLegend	Cat#141702; RRID: AB_10900233
Purified anti-mouse/rat/human FOXP3 antibody (150D)	BioLegend	Cat#322002; RRID: AB_439746
CD103 (Integrin alpha E) monoclonal antibody (2E7), eBioscience	Invitrogen	Cat#14-1031-82; RRID: AB_467409
Mouse CCR2 antibody (475301)	R&D Systems	Cat#MAB55381; RRID: AB_2749828
BD Pharmingen purified mouse anti-mouse RORγt (Q31-378)	BD Biosciences	Cat#562663; RRID: AB_2687844
Arginase 1 monoclonal antibody (GT5811)	Invitrogen	Cat#MA5-31577; RRID: AB_2787204
BD Pharmingen purified rat anti-mouse CD16/CD32 (mouse BD Fc Block)	BD Biosciences	Cat#553142

**Chemicals, peptides, and recombinant proteins**		

Cell-ID Intercalator-Ir—125 μM	Standard BioTools	Cat#201192A
Cell-ID Cisplatin, 100 μL	Standard BioTools	Cat#201195
Tuning solution—250 mL	Standard BioTools	Cat#201072
Antibody stabilizer PBS 50 mL	CANDOR	Cat#131050
HRP-Protector	CANDOR	Cat#222050
Solution TCEP Bond-Breaker, pH neutral	Thermo Scientific	Cat#77720
DMEM, low glucose, pyruvate	Gibco	Cat#31885023
CryoStor CS10	Sigma-Aldrich	Cat#C2874-100ML
RPMI 1640 medium, GlutaMAX supplement	Gibco	Cat#61870010
Trypan blue solution 0.4%	Sigma-Aldrich	Cat#T8154-100ML
DPBS (1×), no calcium, no magnesium	Gibco	Cat#14190094
VERSYLENE FRESENIUS sterile water	Fresenius	Cat#B230531
Fetal bovine serum (South America) - 500 mL	Dutscher	Cat#S1810-500
Accutase cell detachment solution	BioLegend	Cat#423201
Recombinant IFN-γ	PeproTech	Cat#315-05
Lipopolysaccharides from *Escherichia coli* O55:B5	Sigma-Aldrich	Cat#L2880
Recombinant IL-4	PeproTech	Cat#214-14
Recombinant IL-13	PeproTech	Cat#200-13
Dexamethasone	Santa Cruz	Cat#sc-29059
Recombinant IL-10	PeproTech	Cat#200-10
Maxpar cell staining buffer—500 mL	Standard BioTools	Cat#201068
Maxpar cell acquisition buffer—200 mL	Standard BioTools	Cat#201240
EQ four element calibration beads—100 mL	Standard BioTools	Cat#201078
Maxpar PBS (500 mL)	Standard BioTools	Cat#201058
Maxpar Fix and Perm buffer—100 mL	Standard BioTools	Cat#201067
RBC lysis buffer (10×)	BioLegend	Cat#420301
16% formaldehyde (p/v) Pierce without methanol	Thermo Scientific	Cat#28906
DNase I from bovine pancreas	Roche	Cat#11284932001
Collagenase D from *Clostridium histolyticum*	Roche	Cat#COLLD-RO

**Critical commercial assays**		

Maxpar X8 antibody labeling kit, 154Sm—4 Rxn	Standard BioTools	Cat#201154A
Maxpar X8 antibody labeling kit, 163Dy—4 Rxn	Standard BioTools	Cat#201163A
Maxpar X8 antibody labeling kit, 169Tm—4 Rxn	Standard BioTools	Cat#201169A
Maxpar X8 antibody labeling kit, 173Yb—4 Rxn	Standard BioTools	Cat#201173A
Maxpar X8 antibody labeling kit, 147Sm—4 Rxn	Standard BioTools	Cat#201147A
Maxpar X8 antibody labeling kit, 156Gd—4 Rxn	Standard BioTools	Cat#201156A
Maxpar X8 antibody labeling kit, 158Gd—4 Rxn	Standard BioTools	Cat#201158A
Maxpar X8 antibody labeling kit, 160Gd—4 Rxn	Standard BioTools	Cat#201160A
Maxpar X8 antibody labeling kit, 171Yb-4 Rxn	Standard BioTools	Cat#201171A
Maxpar X8 antibody labeling kit, 172Yb-4 Rxn	Standard BioTools	Cat#201172A
Maxpar X8 antibody labeling kit, 174Yb-4 Rxn	Standard BioTools	Cat#201174A
Maxpar X8 antibody labeling kit, 167Er-4 Rxn	Standard BioTools	Cat#201167A
Maxpar X8 antibody labeling kit, 166Er-4 Rxn	Standard BioTools	Cat#201166A
Maxpar X8 antibody labeling kit, 155Gd-4 Rxn	Standard BioTools	Cat#201155A
Maxpar X8 antibody labeling kit, 168Er-4 Rxn	Standard BioTools	Cat#201168A
Maxpar X8 antibody labeling kit, 175Lu-4 Rxn	Standard BioTools	Cat#201175A
Maxpar MCP9 antibody labeling kit, 116Cd—4 Rxn	Standard BioTools	Cat#201111A
eBioscience Foxp3/transcription factor staining buffer set	Invitrogen	Cat#00-5523-00
Dead cell removal kit	Miltenyi Biotec	Cat#130-090-101

**Experimental models: Cell lines**		

RAW264.7 mouse macrophage cell line	ATCC	Cat#ATCC TIB-71

**Experimental models: Organisms/strains**		

Adult female C57BL/6JRj mice older than 8 weeks	Janvier Labs	Cat#SC-C57J-F

**Software and algorithms**		

OMIQ	Domatics	https://www.omiq.ai/

**Other**		

Amicon Ultra-0.5, membrane Ultracel-3, PMNL 3 kD	Merck	Cat#UFC500396
Amicon Ultra-0.5, membrane Ultracel-50, PMNL 50 kD	Merck	Cat#UFC505096
Amicon Ultra-0.5, membrane Ultracel-100, PMNL 100 kD	Merck	Cat#UFC510008
gentleMACS C tubes	Miltenyi Biotec	Cat#130-093-237
Falcon cell strainers 40 μm	Falcon	Cat#352340
MS columns	Miltenyi Biotec	Cat#130-042-201
OctoMACS separator	Miltenyi Biotec	Cat#130-042-109
MACS MultiStand	Miltenyi Biotec	Cat#130-042-303
gentleMACS dissociator	Miltenyi Biotec	Cat#130-093-235
Helios, a CyTOF system	Standard BioTools	N/A
Filter tips, 200 μL Biosphere	Sarstedt	Cat#70.3031.255
Filter tips, 10 μL Biosphere	Sarstedt	Cat#70.1115.210
Pipette tips HRC UNV 1,000 μL	Mettler Toledo	Cat#768C/8
SafeSeal reaction tube, 0.5 mL, PP	Sarstedt	Cat#72.704
Microtube SafeSeal, 1.5 mL, PP	Sarstedt	Cat#72.706
Falcon 5 mL round bottom polystyrene test tube	Corning	Cat#352052
Falcon 50 mL high clarity PP centrifuge tube	Corning	Cat#352070
Falcon 15 mL high clarity PP centrifuge tube	Corning	Cat#352096
Centrifuge 5427 R	Eppendorf	Cat#5429000010
MyFuge 12 mini centrifuge	Benchmark Scientific	Cat#C1012
NanoDrop 1000 spectrophotometer	Thermo Scientific	N/A
JULABO water bath	JULABO	N/A

## Materials and equipment

**Table:** RPMI medium-10% FBS

Reagent	Final concentration	Amount
RPMI 1640 Medium, GlutaMAX	N/A	449 mL
FBS	10%	50 mL
Penicillin/Streptomycin	0.2% (10000 U/mL; 10 μg/mL)	1 mL
**Total**	**N/A**	**500 mL**

Sore at 4°C for 12 months

**Table:** DMEM medium-10% FBS

Reagent	Final concentration	Amount
DMEM low glucose, pyruvate	N/A	449 mL
FBS	10%	50 mL
Penicillin/Streptomycin	0.2% (10000 U/mL; 10 μg/mL)	1 mL
**Total**	**N/A**	**500 mL**

Sore at 4°C for 12 months.

**Table:** Pro-inflammatory M1 macrophage polarization solution

Reagent	Final concentration	Amount
DMEM medium-10% FBS	N/A	994 μL
LPS (20 μg/mL)	100 ng/mL	5 μL
IFN (20 μg/mL)	20 ng/mL	1 μL
**Total**	**N/A**	**1 mL**

Prepare the day of experiment and leave at 4°C.

**Table:** Alternative M2a macrophage polarization solution

Reagent	Final concentration	Amount
DMEM medium-10% FBS	N/A	998 μL
IL-4 (20 μg/mL)	20 ng/mL	1 μL
IL-13 (20 μg/mL)	20 ng/mL	1 μL
**Total**	**N/A**	**1 mL**

Prepare the day of experiment and leave at 4°C.

**Table:** Alternative M2c macrophage polarization solution

Reagent	Final concentration	Amount
DMEM medium-10% FBS	N/A	994 μL
Dexamethasone (10 μM)	50 nM	5 μL
IL-10 (20 μg/mL)	20 ng/mL	1 μL
**Total**	**N/A**	**1 mL**

Prepare the day of experiment and leave at 4°C.

• 30 mg/mL collagenase D: add 30 mg collagenase D in 1 mL 1× PBS.

 [Store at −20°C for up to one week]

• 10 mg/mL DNase I: add 10 mg DNase I in 1 mL dH_2_O.

 [Store at −20°C for up to one month]

**Table:** Digestion solution

Reagent	Final concentration	Amount
Collagenase D (30 mg/mL)	2 mg/mL	267 μL
DNase I (10 mg/mL)	0.1 mg/mL	40 μL
RPMI supplemented per 10% FBS	N/A	3.693 mL
**Total**	**N/A**	**4 mL**

Prepare the day of experiment and leave at 4°C.

## Step-by-step method details

### Preparation of single-cell suspension from mouse tissues and peritoneal lavage

**Timing: 5 h**

Cell suspension preparation is an important step in a mass cytometry protocol, and the tissue dissociation method must be adapted to each tissue to enable complete dissociation while preserving cellular epitopes.^5^ This step describes how to digest lung and spleen tissues to obtain a high-viability single-cell suspension, as well as how to prepare a single-cell suspension from intraperitoneal lavages.

***Note:*** Before harvesting the organs, intravascular lavage is recommended to avoid contamination with blood cells. troubleshooting 2.

1. Preparation of lung cell suspension.
  **CRITICAL:** Work with freshly isolated lung tissue and not with thawed tissue. troubleshooting 3.
  a. Dissociate lung samples.
    i. Place lung sample in petri dish containing RPMI medium supplemented with 10% of FBS on ice.
    ii. Cut the sample into small pieces using scalpel (2–3 mm).
    iii. Add 4 mL of digestion solution (as described in the “materials and equipment” section) in gentleMACS C-tubes on ice, and place minced samples in this tube.
    iv. Insert C-tubes in the gentleMACS dissociator and start the program set to 165 rounds/run for 37 s.
    v. Incubate tubes at 37°C for 15 min in water bath and shake it every 5 min.
    vi. Insert C-tubes in the gentleMACS dissociator and start the program set to 2079 rounds/run for 38 s.
  b. Filter the lysate in 40-μm cell strainer placed on 50 mL Falcon tube and press gently using the plunger of a syringe to complete dissociation.
  c. Rinse the cell strainer with 5 mL of 1× PBS supplemented with 2% of FBS.
  d. Centrifuge at 400 × *g* for 10 min at 4°C.
  e. Lyse red blood cells.
    i. Prepare 5 mL of 1× RBC lysis buffer per sample by diluting the 10× RBC lysis buffer (BioLegend) to 1:10 in Milli-Q water.
    ii. Resuspend cell pellet with 5 mL of 1× RBC lysis buffer.
    iii. Incubate on a rotary shaker for 5 min at 4°C in the dark.
    iv. Stop lysis by adding 10 mL of 1× PBS supplemented with 2% of FBS.
    v. Centrifuge at 400 × *g* for 10 min at 4°C and discard supernatant.
    vi. Add 3 mL of 1× PBS supplemented with 2% of FBS for a second wash.
    vii. Centrifuge at 400 × *g* for 10 min at 4°C and discard supernatant.
      **Alternative:** Lung samples can be dissociated using other enzymatic digestion cocktails, such as dispase, hyaluronidase, or collagenase IV, or through mechanical digestion using other homogenizer.
  f. Sort dead cells on magnetic column. troubleshooting 4
    ***Note:*** The beads and buffers used to sort dead cells on the magnetic columns are part of the Dead cell removal kit (Miltenyi).
    ***Optional:*** Cells could be count at this step to evaluate the bead volume to add for dead cell sorting (100 μL of beads for up to 10 million of dead cells).
    ***Note:*** 15 ± 5 millions of total cells could be are obtained after entire right lung lobe dissociation with 60 ± 10% cell viability (determined using trypan blue staining).
    i. Add 100 μL of beads from the Dead cell removal kit and resuspend gently using a pipette.
    ii. Incubate at room temperature for 15 min.
    iii. Prepare 3 mL of 1× binding buffer (Dead cell removal kit) per sample by diluting 10× binding buffer to 1:10 in distilled sterile water.
    iv. Add 400 μL of binding buffer 1× on cell suspension after incubation.
    v. Insert MS columns on the separator OctoMACS fixed to a magnetic rack and place 15 mL Falcon tubes under the column.
      ***Note:*** The choice of column type depends on the number of cells obtained and cell viability. In this protocol, MS columns are used because less than 10 million dead cells are obtained. If LS columns are chosen, adapt the protocol using the manufacturer’s recommendations of Dead cell removal kit (Miltenyi, Cat#130-090-101: link).
    vi. Place a 40 μm cell strainer on the top of the column and add 500 μL of 1× binding buffer to humidify cell strainer and column.
    vii. Add cell suspension on the 40 μm cell strainer placed on the column to filter it and to sort dead cells on the magnetic column.
    viii. Wash the cell strainer and the column by adding 500 μL of 1× binding buffer and wait until all the buffer has passed through the column.
    ix. Wash the column 3 more times with 500 μL of 1× binding buffer.
    x. Recover the 15 mL Falcon tubes containing the live cell suspension.
    xi. Centrifuge at 400 × *g* for 5 min at 4°C and discard the supernatant.
      ***Note:*** The cell viability percentage obtained after dead cell sorting is around of 90 ± 5%.
2. Preparation of spleen cell suspension.
  a. Place the tissue in 40 μm cell strainer placed on 50 mL Falcon tube with 1 mL of 1× PBS supplemented with 2% of FBS.
  b. Crush the tissue using the plunger of a syringe.
  c. Rinse the cell strainer with 4 mL of 1× PBS supplemented with 2% of FBS.
  d. Centrifuge at 400 × *g* for 10 min at 4°C.
  e. Discard the supernatant.
  f. Lyse red blood cells as described for the lung samples (Step 1, sub-step e).

***Note:*** After entire spleen dissociation (healthy mice), 46 ± 18 millions of total cells could be obtained with a mean viability of 80 ± 10%.

***Note:*** Spleen tissues can be cryopreserved using a CryoStor CS10 solution (Sigma-Aldrich, Cat#C2874-100ML) according to the recommendations. If the cell viability is lower than 80%, dead cells can be sorted on magnetic column following the protocol above (Step 1, sub-step f).

3. Preparation of cells from peritoneal lavage done with 10 mL of 1× PBS supplemented with 2% of FBS.
  a. Centrifuge at 400 × *g* for 10 min at 4°C.
  b. Discard the supernatant.
  c. Lyse red blood cells as described for the lung samples (Step 1, sub-step e).

***Note:*** In a peritoneal lavage performed with 10 mL of PBS, the mean number of cells recovered is 5 ± 1.5 million for healthy mice and 16 ± 7 million for infected mice.

***Note:*** The cells recovered in peritoneal lavages can be cryopreserved in FBS solution containing 10% of DMSO.

4. Resuspend cells in RPMI medium and mix an equal volume of the cell suspension with a 0.4% trypan blue solution.
5. Count cells on Malassez chamber.

### Cell staining

**Timing: 5 h**

Spleen, lung and intraperitoneal lavage cells are stained with the viability marker Cisplatin that bind to nuclear proteins when cell membrane is permeable. Cells are labeled with a panel composed of 37 antibodies allowing the recognition of 30 surface markers and 7 intracellular markers. For detection of cellular events by the mass cytometer, cells are stained with Iridium, a DNA intercalant.

**CRITICAL:** To avoid metal contaminations, only pipette tips with filter and non-autoclaved materials should be used for this protocol. troubleshooting 1.

***Note:*** Significant cell loss (up to 50%) can occur during the labeling protocol due to the numerous washing steps, especially from the fixation/permeabilization step for intracellular labeling (Step 15). The following protocol is adapted for the staining of up to 3 million of cells in order to obtain at least 1.5 million cells at the end of labeling.

***Optional:*** 15 mL Falcon tubes can be used for staining protocol because they allow to better recover the cell pellet. Cytometry tubes or 3 mL Eppendorf tubes can also be used.

6. Prepare surface and intracellular antibody cocktails in Eppendorf tubes on ice separately using the antibody dilutions selected during titrations, in a final volume of 50 μL.
7. Take the required volume from the cellular suspension obtained in step 5 to have 3 million cells.
8. Add the adequate volume of Maxpar PBS supplemented with 2% of FBS to final volume of 1.5 mL.
9. Centrifuge at 400 × *g* for 5 min at 4°C and discard the supernatant.
10. Add 1.5 mL of Maxpar PBS supplemented with 2% of FBS.
11. Centrifuge at 400 × *g* for 5 min at 4°C and discard the supernatant.
12. Stain cells with Cisplatin for detection of live/dead cells.
  a. Dilute the 5 mM Cisplatin stock solution to 1:1000 by adding 1 μL of Cisplatin in 1 mL of Maxpar PBS for an intermediate concentration of 5 μM.
  b. Prepare 250 μL of 0,5 μM Cisplatin solution per sample by diluting the 5 μM Cisplatin solution to 1:10 in Maxpar PBS.
  c. Add 250 μL of the 0.5 μM Cisplatin solution on cells and resuspend using a pipette.
  d. Incubate cell suspension with Cisplatin at room temperature for 5 min.
  e. Quench the Cisplatin by adding 5× volume of Maxpar Cell Staining Buffer in each tube.
  f. Centrifuge at 400 × *g* for 5 min at 4°C and discard supernatant.
  g. Resuspend cells in 1 mL of Maxpar PBS to wash.
  h. Centrifuge at 400 × *g* for 5 min at 4°C and discard supernatant.
13. Block Fc receptors.
  a. Prepare 100 μL of FcBlock solution per sample (3 million cells) by diluting 6 μL of 0.5 mg/mL FcBlock stock solution in 94 μL of Maxpar Cell Staining Buffer.
    ***Note:*** 1 μg of FcBlock per million cells is required in a final volume of 100 μL per tube.
  b. Add 100 μL of diluted FcBlock solution on cells and resuspend using a P100 pipette.
  c. Incubate cell suspension with FcBlock for 10 min at room temperature.
  d. Wash cells by adding 1 mL of Maxpar cell staining buffer in the tube containing cells in FcBlock solution.
  e. Centrifuge at 400 × *g* for 5 min at 4°C and discard supernatant by pipetting.
14. Stain cells with surface antibody cocktail.
  a. Resuspend cell pellet with 40 μL of Maxpar cell staining buffer to have a final volume of 50 μL (10 μL of residual volume).
  b. Add 50 μL of the surface antibody cocktail.
  c. Mix gently using a pipette and spin for 5 s using a microcentrifuge.
  d. Incubate for 30 min at room temperature. After 15 min of incubation, resuspend gently using a pipette.
  e. Wash cells by adding 3 mL of Maxpar Cell Staining buffer.
  f. Centrifuge at 400 × *g* for 5 min at 4°C and discard supernatant.
15. Fix and permeabilize cells using the Foxp3/Transcription Factor Staining Buffer Set.
  ***Note:*** FixPerm concentrate, Diluent and 10× Permeabilization buffer are part of the Foxp3/Transcription Factor Staining Buffer Set.
  a. Fix cells.
    i. Prepare 3 mL of diluted FixPerm solution per sample (3 million cells) by diluting the FixPerm concentrate to 1:4 in the kit Diluent.
    ii. Add 3 mL of diluted FixPerm solution on the 3 million cells and resuspend gently using a pipette.
    iii. Incubate for 30 min at room temperature.
    iv. Centrifuge at 900 × *g* for 10 min at 4°C and discard supernatant.
      **CRITICAL:** Take care when removing the supernatant, as the cell pellet becomes transparent and slippery at this step.
    v. Prepare 6,5 mL of 1× permeabilization buffer per sample by diluting the 10× Permeabilization buffer to 1:10 in Milli-Q water.
    vi. Wash cells by adding 2 mL of 1× permeabilization buffer.
    vii. Centrifuge at 900 × *g* for 10 min at 4°C and discard supernatant by pipetting.
  b. Permeabilize cells.
    i. Add 40 μL of 1× permeabilization buffer.
    ii. Incubate for 15 min at room temperature.
16. Stain cells with intracellular antibody cocktail.
  a. Add 50 μL of the intracellular antibody cocktail and resuspend gently using a pipette.
  b. Incubate for 30 min at room temperature. After 15 min of incubation, resuspend gently using a pipette.
  c. Add 2 mL of 1× permeabilization buffer buffer (Foxp3/Transcription Factor Staining Buffer Set) for a first wash.
  d. Centrifuge at 900 × *g* for 10 min at 4°C and discard supernatant.
  e. Repeat these two latest steps to wash cells a second time.

**CRITICAL:** At the end of the second wash, remove carefully all the residual volume to avoid diluting the formaldehyde in the next step.

17. Fix cells with formaldehyde.
  a. Prepare 1 mL of 2.5% formaldehyde solution per sample by diluting 16% formaldehyde stock solution to 1:6.4 in Maxpar PBS.
  b. Add 1 mL of 2.5% formaldehyde solution and resuspend gently using a pipette.
  c. Incubate for 10 min at room temperature.
  d. Centrifuge at 900 × *g* for 10 min at 4°C and discard supernatant.
18. Stain cells with Iridium.
  a. Prepare 1 mL of 0.125 μM iridium solution per sample by diluting 125 μM iridium stock solution to 1:1000 in Maxpar Fix and Perm buffer.
  b. Add 1 mL of 0.125 μM Iridium solution on cell pellet and resuspend cells gently.
  c. Incubate for 1 h at room temperature or at 4°C for a night.

**Pause point:** Cells can remain in the iridium solution for up to 48 h at 4°C. troubleshooting 5.

### Sample acquisition

**Timing: 1 h per sample.**Troubleshooting 6.

19. Wash cells by adding 2 mL of Maxpar Cell Staining buffer.
20. Centrifuge at 800 × *g* for 5 min at 4°C and discard supernatant.
21. Wash twice by adding 2 mL of Maxpar Cell Acquisition buffer.
22. Centrifuge at 800 × *g* for 5 min at 4°C and discard supernatant.
23. Prepare 3 mL per sample (3 million cells) of 0.1× EQ beads by diluting 1 part beads to 9 parts of Maxpar Cell Acquisition buffer.
24. Before data acquisition, completely resuspend cells to the maximum recommended cell concentration of 1 × 10^6^ cells/mL in 0.1× bead solution.
25. Filter the cells through a 40 μm cell strainer.
26. Start the Helios mass cytometer and leave it for 20 min for plasma stabilization.
27. Use Tuning Solution (Standard Biotools) to optimize the instrument according to the manufacturer’s recommendations in the Tune tab.
28. Check these parameters: an extra threshold for successful tuning, Dual Slope values for Cs and Tm: 0.03 +/−0.003; Mean Dual Count Tb value: >600000; RSD (Dual) values for Tb, Cs, La, Tm, Ir: <3%; Resolution (mass1): >400; R2: >0.8; Oxide ratio (M1/M2): < 0.03.
29. Acquire events using the appropriate acquisition template and normalize FCS files utilizing CyTOF Software 7.0 8493 Standard BioTools, California, USA.
  a. Open the Acquire Tab.
  b. Open the Experiment Manager Tab select or create your template, which corresponds to panel table (label and corresponding marker columns), and select Event mode.
  c. Open Stop at Tab select event, we acquire minimum of 500,000 events per files.
  d. Run your samples in previous mode and wait until the number of events per second is stable before record.

## Expected outcomes

**Figure 4:**
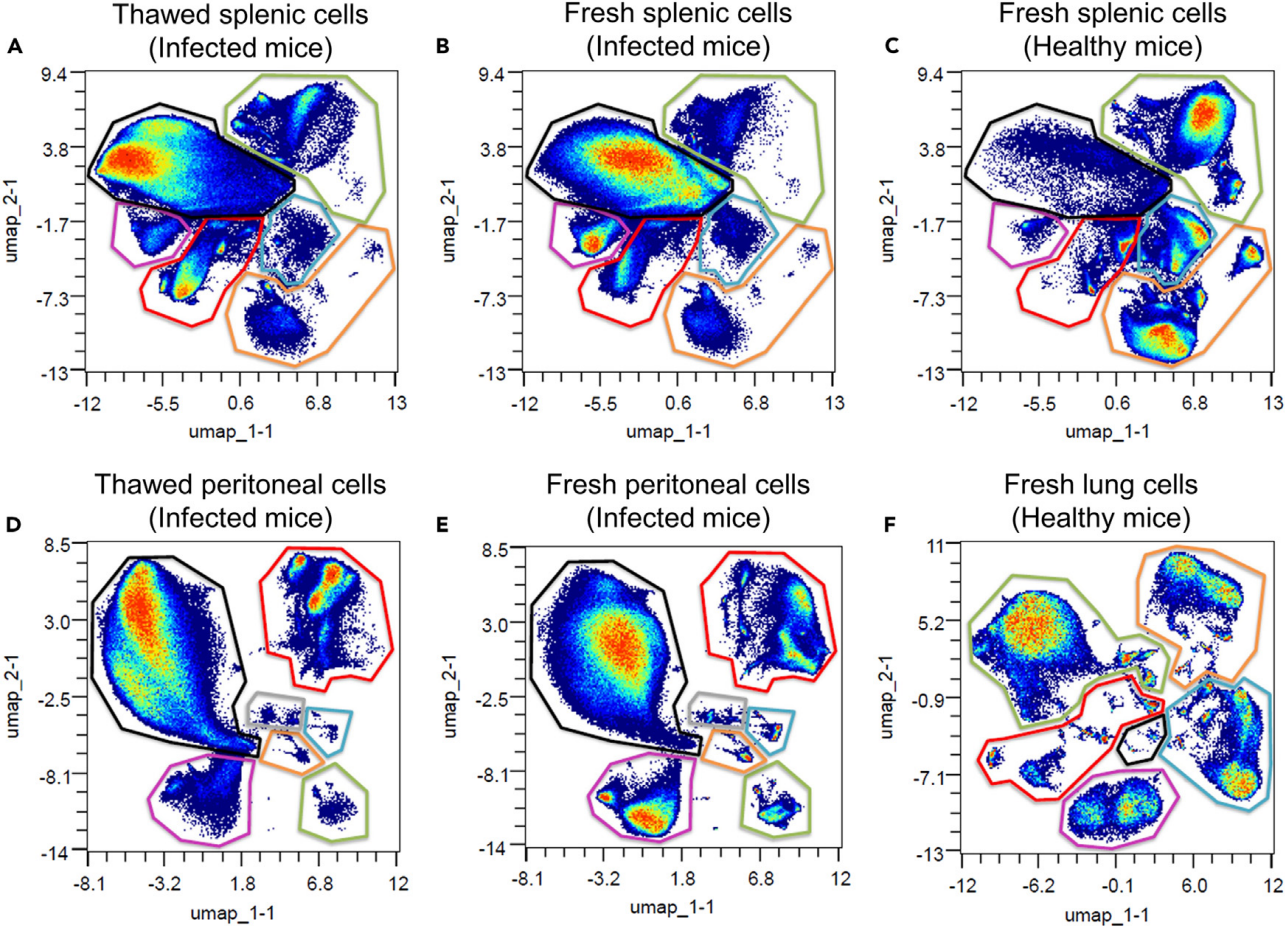
UMAP plots of mouse cells analyzed by mass cytometry UMAP is a dimensionality reduction technique, which allows summarizing *n* dimensions data into two dimensions. These graphs show the UMAP plots for cells isolated from thawed (A) and fresh (B) spleen of mice infected with *Echinococcus multilocularis*, cells isolated from fresh spleen of uninfected mouse (C), thawed (D) and fresh (E) peritoneal cells of mice infected with *E. multilocularis*, and cells isolated from fresh lungs of uninfected mouse (F). Gates show B cells (green), T CD4^+^ cells (orange), T CD8^+^ cells (blue), monocytes/macrophages/dendritic cells (red), eosinophils (pink), neutrophils and precursors (black) and undetermined populations (gray). Thawed cells (especially myeloid cells) had modified expression of some markers, compared to fresh cells (A vs. B, and D vs. E, respectively). This modified slightly their UMAP distribution and can interfere with analysis and interpretation process.

This protocol allows to phenotype cell populations isolated from lung, spleen and peritoneal lavage. The markers included in this panel enable the identification of lymphoid and myeloid cells determined by supervised or unsupervised analysis using cell markers described in the literature, which depend on the tested tissue and pathological condition (Table 2; Figure 4). The analysis was performed using the Omiq software from Dotmatics (www.omiq.ai, www.dotmatics.com, 2023) and applying usual quality controls for mass cytometry data.^6^

**Table 2:** Summary table of markers expressed for each cell type in mouse lungs, spleen and peritoneal lavage

Cell populations	Lungs	Spleen	Peritoneal lavages
Macrophages	CD45^+^ F4/80^+^ MERTK^+^ CD64^+^ CD172a^+^		
Tissue subtypes	• Alveolar macrophages (AM): CD169^+^ CD170^+^ CD68^hi,^ CD206^hi^ CD11c^+^ CD11b^+/−^ • Interstitial macrophages (IM): CD11b^+^ I-A/I-E^+^ CD11c^+/−^ CD206^+/−^ CCR2^+/−^	• Red pulp macrophages: CD11b^lo^ F4/80^hi^CD169^-^ • Marginal zone macrophages: CD11b^hi^F4/80^lo^ CD169^lo/-^ • Marginal metallophilic macrophages : CD11b^hi^F4/80^-^CD169^+^	• Large peritoneal macrophages (LPM): I-A/I-E^-^CCR2^-^ • Small peritoneal macrophages (SPM): I-A/I-E^+^CCR2^+^
Polarization markers	Arginase 1, iNOS, CD204, CD115, Trem2		
Monocytes	CD45^+^ CX3CR1^+^ CD11b^+^ CD172a^+^ CCR2^+^		
Classical	Ly6C^hi^ CD43^lo^		
Non-classical	Ly6c^lo^ CD43^hi^		
Granulocytes	CD45^+^ CD11b^+^ CD24^+^		
Neutrophils	Ly6G^+^		
Eosinophils	CD170^+^F4/80^+^		
Dendritic cells	CD45^+^ CD11c^+^ CD24^+^ CD209^+/−^		
Conventional 1	CD103^+^ I-A/I-E+^+^		
Conventional 2	CD11b^+^ CD172a^+^ I-A/I-E^+^		
Plasmacytoid	SiglecH^+^		
Natural killers	CD45^+^ CD11b^+^ CD161^+^		
Lymphocytes	CD45^+^		
B cells	CD19^+^ B220^+^ I-A/I-E^+^		
CD4+ T cells	CD3e^+^, CD4^+^ • T helper 17 cell: RORγT^+^ • Regulatory T cell: FoxP3^+^		
CD8+ T cells	CD3e^+^ CD8a^+^		
Structural cells	CD45^-^		
Epithelial cells	CD326^+^ Gp-38^+/−^ CD24^+/−^		
Endothelial cells	CD31^+^

**Figure 5:**
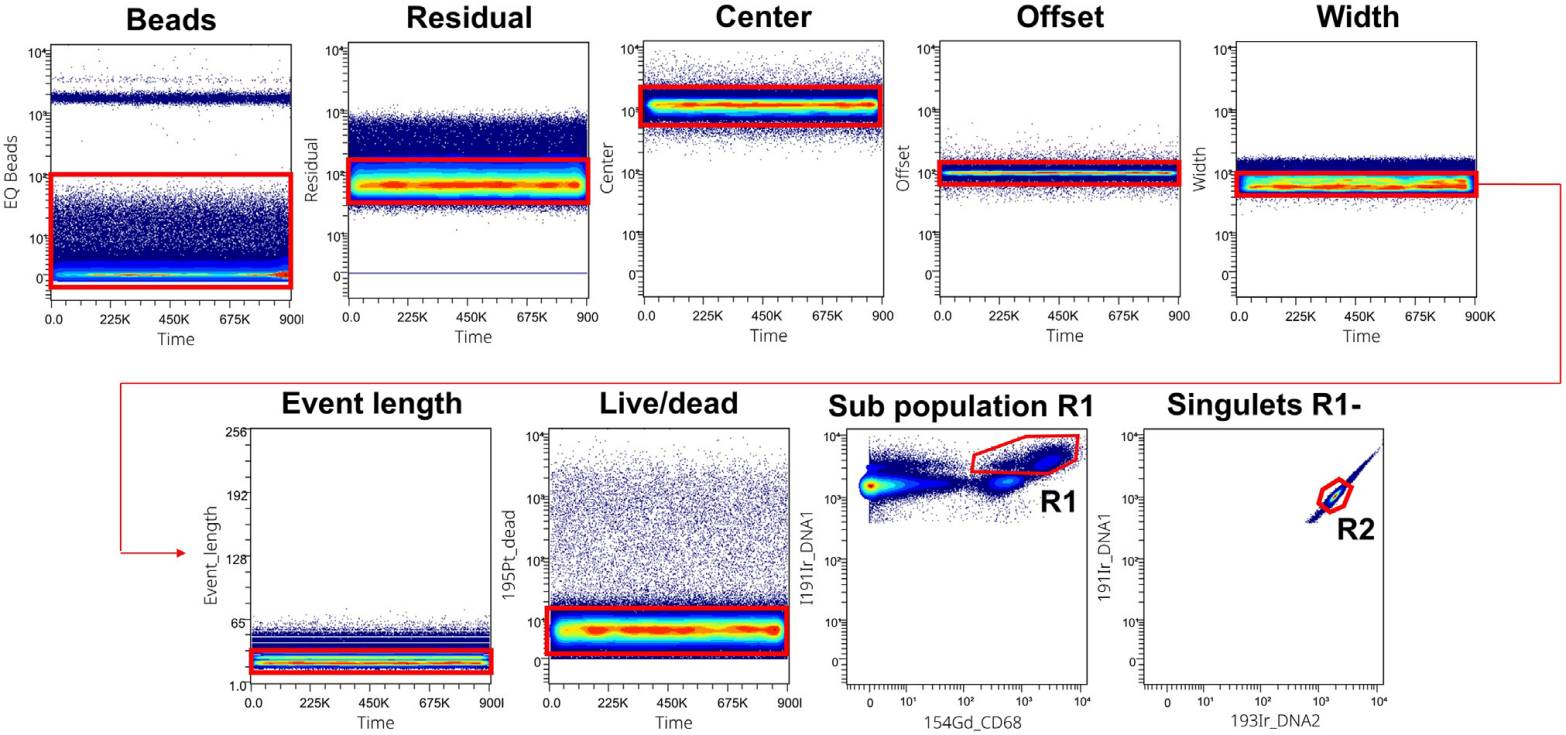
Data cleaning strategy The gating strategy first eliminated the normalization beads and then the residual elements and non-Gaussian pulses were eliminated based on the Gaussian distribution of the Residual, Center, Offset, Width and Event length parameters. The subpopulation of macrophages incorporating a greater quantity of iridium (R1) was gated and the elimination of doublets was carried out on events excluding R1 (R2).

For the data cleaning strategy, we followed the strategy shown by C.B Bagwell et al.^7^ This gating strategy, utilizing Gaussian discrimination parameters, is applied to eliminate undesired elements post normalization such as beads, residual elements and non-Gaussian pulses. The dead cells incorporating Cis-platin are then removed. Finally, the doublets with the highest iridium levels are eliminated. Results showed that a sub-population of macrophages appeared to be highly incorporated into the iridium and if doublets were removed, this population was lost. This observation was also mentioned by Brian H. Lee and Adeeb H. Rahman.^8^ The strategy is therefore to keep this population named R1 and to eliminate the doublets on the R1- events. The R1 and R2 events are then merged for the final analysis (Figure 5).

**Figure 6:**
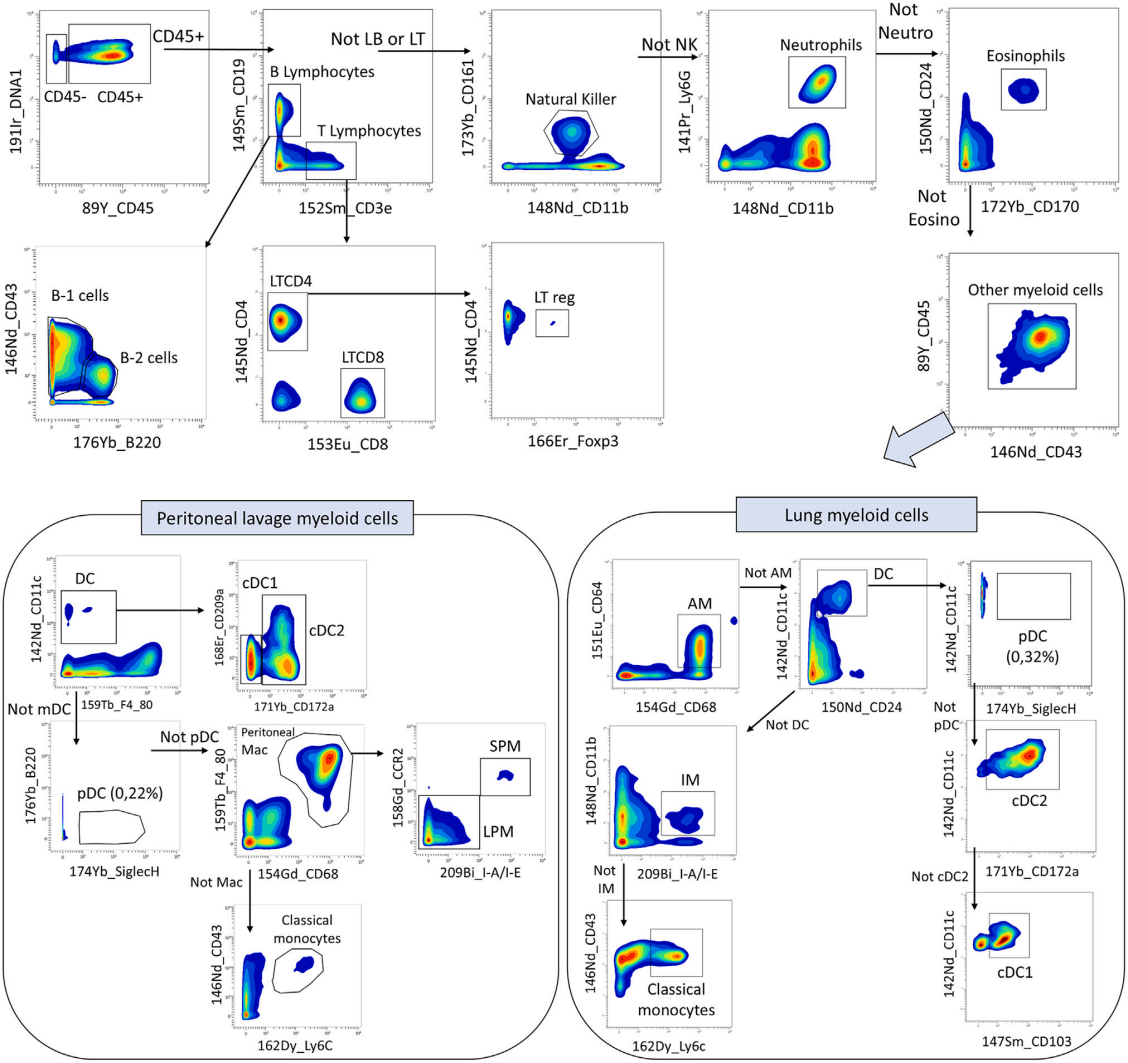
Proposed gating strategy for identifying key murine tissue cell populations in both peritoneal lavage and lung tissue Mouse lung cells were isolated and labeled with the panel of 37 markers proposed in this protocol. After cell acquisition using the Helios mass cytometer and data cleaning, cells were identified using a gating strategy on the OMIQ software. The CD45- cells and CD45+ immune cells were first identified. We then identify B cells (CD19^+^) and among them B1 and B2 subpopulations, T cells (CD3^+^) and among them LTCD8+ and LTCD4+ and notably LTreg (Foxp3^+^), Natural killers (NK1.1^+^), neutrophils (Ly6G^+^) and eosinophils (CD24^+^CD170^+^). The remaining population, called "other myeloid cells", consisted mainly of monocytes, macrophages and dendritic cells (DC). In peritoneal lavage, the two populations of conventional DC, cDC1 and cDC2, were identified among mDC (CD11c^+^) based on CD172a and CD209a staining. After exclusion of plasmacytoid DC (pDC) (SiglecH^+^), two subpopulations of peritoneal macrophages (PM) (CD68^+^ F4/80^+^) were identified, the small PM (SPM)(I-A/I-E^hi^ and CCR^hi^) and the large PM (LPM). Finally, we identified classical monocytes based on Ly6c^hi^ and CD43^lo^ staining. In lung tissue, we first identified alveolar macrophages (AM) (CD64^+^ CD68^+^) and after DC exclusion, we identified interstitial macrophages (IM) subpopulations (CD11b^+^ I-A/I-E^+^) and classical monocytes (Ly6c^hi^ and CD43^lo^). Among CD11c^+^ dendritic cells, we next identified plasmacytoid DC (pDC) (SiglecH^+^) and the two subpopulations of conventional DC named cDC1 (CD103^+^) and cDC2 (CD172a^+^).

We propose a gating strategy that identifies B1 and B2 cells, CD8^+^ T cells and CD4^+^ T cells including the regulatory T cells (Treg), NK cells, neutrophils and eosinophils. The remaining population called "other myeloid cells" corresponds mainly to the different populations of monocytes, macrophages and dendritic cells. We next propose two gating strategies for these “other myeloid cells”, one for peritoneal lavage, and one for lung tissue. For both, we identified classical monocytes based on Ly6C and CD43 staining and, among CD11c^+^ dendritic cells, we identified plasmacytoid DC (pDC) and the two subpopulations of conventional DC, named cDC1 and cDC2. Concerning identification of macrophages, a tissue specific gating strategy is required. In peritoneal lavage, we identified, among F4/80^hi^ and CD68^+^ peritoneal macrophages (PM), the small PM (MHCII^hi^ and CCR^hi^) from the large PM subpopulations. In lung tissue, we were also able to identify the alveolar macrophage (AM) and interstitial macrophages (IM) subpopulations (Figure 6).

**Figure 7:**
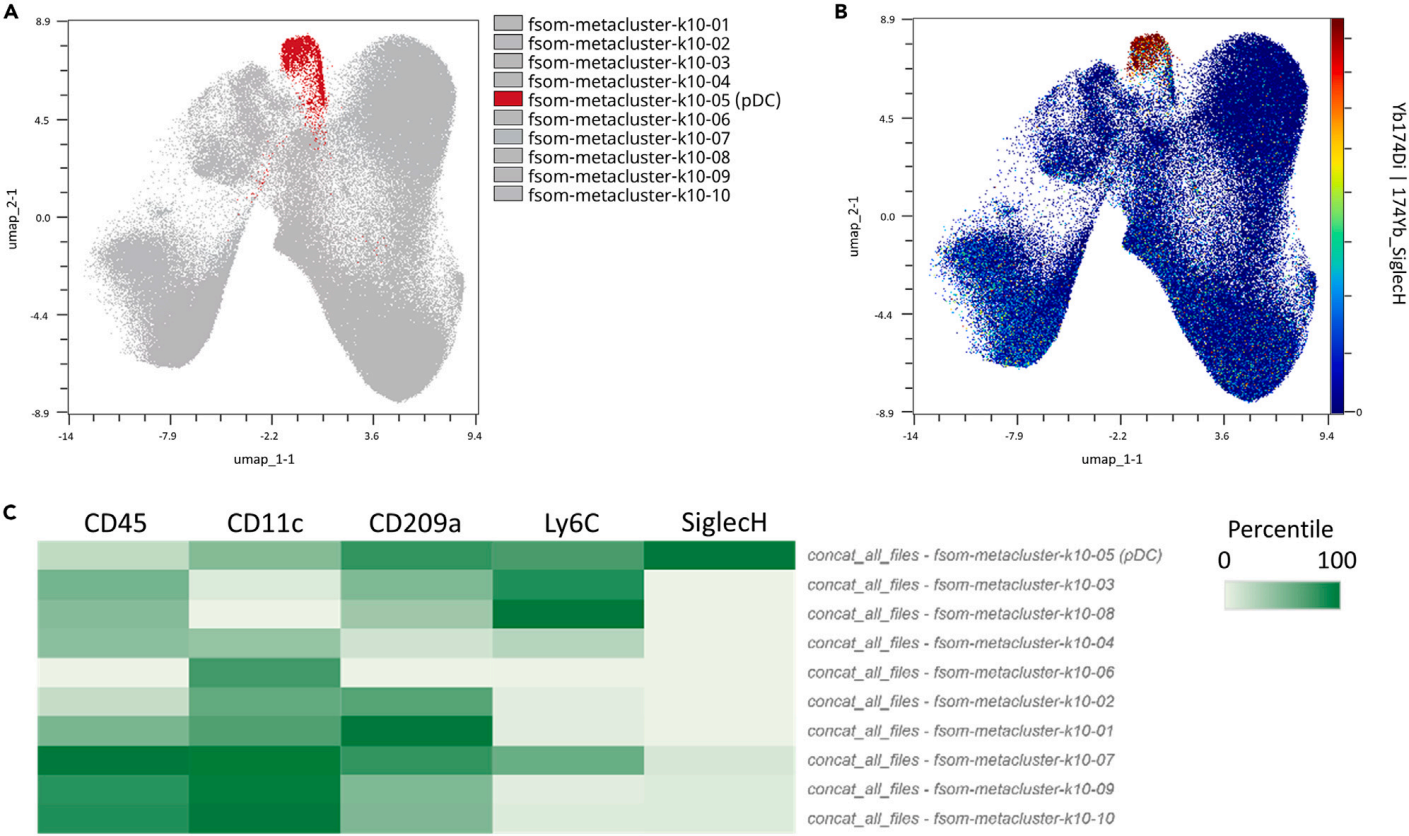
Identification of lung plasmacytoid dendritic cells by unsupervised analysis Flowsom cluster analysis of lung monocytes, macrophages and dendritic cells from merged data of five control mice, the cluster 5 representing pDC (A), and UMAP of the SiglecH expression on these lung cells (B). Heatmap of the median expression value of pDC key markers (CD45, CD11c, CD209a, Ly6C, SiglecH) across the 10 metaclusters identified by Flowsom algorithm (C).

Unsupervised analysis can also be performed using machine-learning. Dimension reduction algorithms such as optSNE or UMAP and clustering algorithms as Flowsom can be used for data visualization and clustering.^9^^,^^10^ As pDC identification was difficult due to a low number of cells (Figure 6), we made an unsupervised analysis to identify them on lung from heathy mice and to prove the pertinence to do both analysis. The Flowsom metacluster 5 represents pDC cell population (Figure 7A) that express Siglec H (Figures 7B and 7C).

We have also demonstrated that this protocol makes it possible to identify lymphoid and myeloid cell populations in pathological conditions as shown data obtained from the spleen of mice infected with a parasite and also in fresh or thawed spleen cells (Figure 4) making this protocol usable for various tissues (healthy versus pathologic) and also for some cryopreserved tissues.

## Limitations

**Figure 8:**
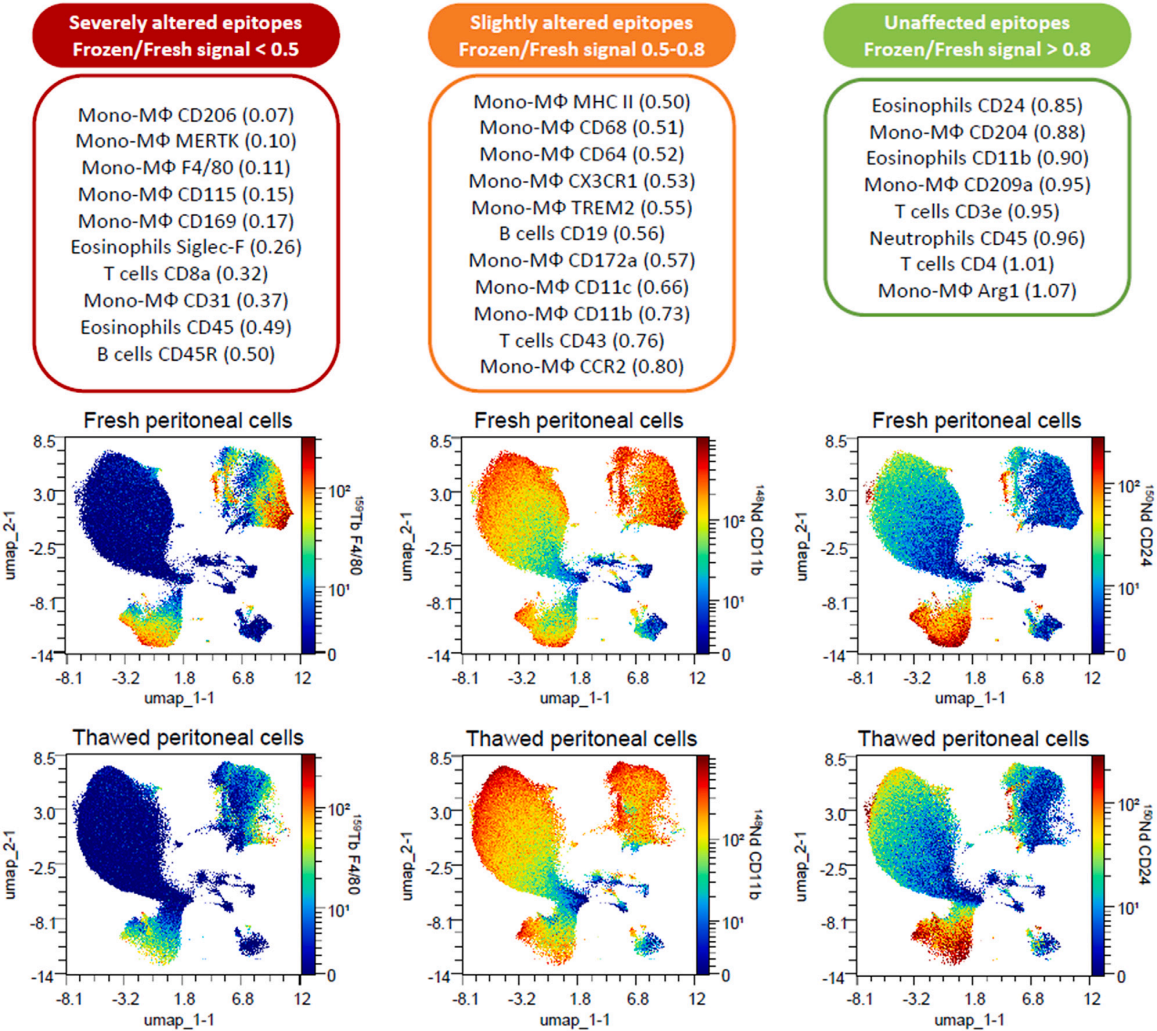
Impact of freezing and thawing process on marker staining intensity Lists of markers (up) for which signal intensity was severely (left), slightly (middle) or not altered (right) by the freezing and thawing process. For each marker/cell population couple, the ratio of frozen on fresh mean intensity signals is shown into brackets. An example among these markers is shown as a UMAP plot with staining intensity in z-axis (bottom). These results are an example based on peritoneal cells from a unique experiment, with one mouse in each group, and could vary depending on the used experimental model, tissues, cellular types and reagents.

This protocol allows the phenotyping of myeloid and lymphoid cells in fresh lung but not in thawed lung samples because of the lower cell viability. We showed the process is applicable to thawed spleen and peritoneal cells. However, some epitopes can be altered (Figure 8), possibly due to changes in marker expression or to lysis of specific populations (e.g., polymorphonuclear cells).

This protocol describes lung and spleen dissociation protocols but mechanical and/or enzymatic dissociation must be adapted according to tissue and pathologies. The proposed panel enables identification of the main lymphoid populations and deeper identification of myeloid populations, but it can be adapted or supplemented depending on the project.

## Troubleshooting

### Problem 1

Metal contaminations and unwanted signals are observed during the analysis of the results.

### Potential solution

Contaminants can arise from various sources, such as autoclaved materials, metal tubes or caps, unsterile water, rubber from syringe filters during tissue digestion, or reagents, tubes or pipette contaminated with environmental dust. To avoid contaminations, ensure that appropriate and clean tubes, equipment, and reagents are used.

### Problem 2

The proportions of some cell populations found in the tissues are not consistent to those found theoretically in literature and seem to be biased by populations frequently found in large quantities in the blood (e.g., monocytes).

### Potential solution

Blood cell contaminations may bias the results and intravascular washing may be necessary. To do this, cut off a piece of the liver and then inject 1× PBS into the right chamber of the heart to eliminate the blood present in the organ vessels.

### Problem 3

The thawing of lung and spleen tissues can result in higher cell mortality rates. If the viability is lower than 60–70%, the DNA release by cells and debris will make the solution sticky which risk to plug the mass cytometer tubing (related to Step 1 and 2).

**Table 3:** Comparison of cell viability percentages between fresh and thawed tissue before and after sorting dead cells

	Fresh lungs	Thawed lungs	Fresh lungs	Thawed lungs
	Before dead cell sorting		After dead cell sorting	
Cell viability studied by trypan blue coloration	66%	40%	90%	32%
Cell viability studied by flow cytometry	58%	45%	96%	-

### Potential solution

**Figure 9:**
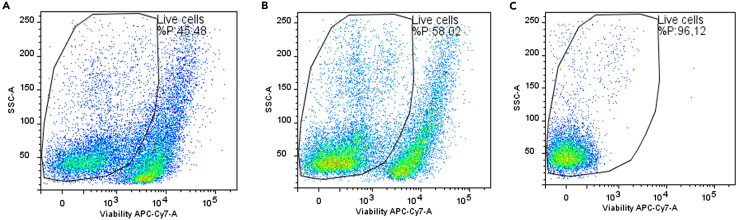
Analysis of cell viability after lung dissociation by flow cytometry Lung cell viability was studied from thawed tissue without dead cell sorting (A) and from fresh tissue before (B) and after dead cell sorting (C).

• Spleen tissues can be stored in CryoStor CS10 solution (Sigma-Aldrich, Cat#C2874-100ML) to limit cell mortality during thawing.
• Do not use a cell strainer larger than 40 μm.
• It is recommended to not use thawed lung tissue but fresh one because of very high mortality rate, even after magnetic column sorting (Figure 9; Table 3).

### Problem 4

The cell yield is low after dead cell sorting on magnetic column because of the DNA release by dead cells, which sticks living and dead cells together (related to Step 1.f.).

### Potential solution

The dead cell removal kit allows dead cells to be sorted from a sample containing up to 50% mortality. The protocol must be modified if the mortality is higher than 50% following manufacturer’s recommendations (Miltenyi Biotec).

• Add DNase I to the cell suspension at concentration of 200 U/ml and incubated for 5 min. If necessary, increase the incubation time.
• Perform a second dead cell sorting to better eliminate dead cells.

### Problem 5

A loss of cells is observed between the end of labeling and acquisition in the mass cytometer.

### Potential solution

The cell fixation is not efficient, the percentage of formaldehyde should be increased to 3%.

### Problem 6

The number of samples is too large to be acquired in the mass cytometer in a single day, increasing the risk of batch effects.

### Potential solution

To avoid batch effects, samples can be run on two consecutive days. In this case, make sure to not acquire samples from the same experimental group on the same day.

## Resource availability

### Lead contact

Further information and requests for resources and reagents should be directed to and will be fulfilled by the lead contact, Valérie Lecureur (valerie.lecureur@univ-rennes.fr).

### Technical contact

Technical questions on executing this protocol should be directed to and will be answered by the technical contact, Valérie Lecureur (valerie.lecureur@univ-rennes.fr).

### Materials availability

This study did not generate new unique reagents.

### Data and code availability

The titration cytometry datasets supporting the current study are available from the corresponding author on request. There are restrictions to the availability of expected outcome datasets because they have not yet been the subject of a scientific article.

## Acknowledgments

This research protocol was funded by “Défis scientifiques” 2022 and 2023, obtained from the University of Rennes, and by the “Groupe Francophone de Recherche sur la Sclérodermie” (AAP 2022). We acknowledge the cytometry flow core facility Hyperion (Brest, France) for its technical assistance and the European subsidy program FEDER Progos RU 000950. We warmly thank the Institut de Parasitologie de l'Ouest, Rennes, France, for financial support (#IPO-2022).

## Author contributions

Conceptualization, supervision, and funding acquisition: V.L., S.D., M.R., and P.H.; sample collection and experiments: L.M., B.A., S.D., V.L., and S.L.G.; acquisition and analyses of the data: P.H. and L.M.; preparation of the figures: B.A. and L.M.; manuscript writing – original draft: L.M., B.A., P.H., S.D., and V.L.; all authors reviewed and edited the final version of the manuscript.

## Declaration of interests

The authors declare no competing interests.
